# Effect of Storage Conditions on Phenolic Profiles and Antioxidant Activity of Litchi Pericarp

**DOI:** 10.3390/molecules23092276

**Published:** 2018-09-06

**Authors:** Mei Deng, Yuanyuan Deng, Lihong Dong, Yongxuan Ma, Lei Liu, Fei Huang, Zhencheng Wei, Yan Zhang, Mingwei Zhang, Ruifen Zhang

**Affiliations:** 1 Sericultural & Agri-Food Research Institute, Guangdong Academy of Agricultural Sciences/Key Laboratory of Functional Foods, Ministry of Agriculture/Guangdong Key Laboratory of Agricultural Products Processing, Guangzhou 510610, China; monaelegant@foxmail.com (M.D.); yuanyuan_deng@yeah.net (Y.D.); dolify@163.com (L.D.); mayongxuan@gdaas.cn (Y.M.); toleiliu@gmail.com (L.L.); hf1311@163.com (F.H.); zhencheng_wei@163.com (Z.W.); zhang__yan_@126.com (Y.Z.); 2 College of Food Science and Technology, Huazhong Agricultural University, Wuhan 430070, China

**Keywords:** litchi pericarp, storage, phenolic compounds, cellular antioxidant activity

## Abstract

Changes of phenolic profiles and antioxidant activity of litchi pericarp during storage at 4 °C for seven days and at room temperature (RT) for 72 h were evaluated in this study. The contents of total phenolic and procyanidin decreased by 20.2% and 24.2% at 4 °C and by 37.8% and 47.8% at RT, respectively. Interestingly, the corresponding reductions of anthocyanins were 41.3% and 73%, respectively. Four phenolic compounds, including epicatechin, procyanidin A_2_, procyanidin B_2_, and quercetin-3-*O*-rutinoside-7-*O*-*α*-l-rhamnosidase were detected in litchi pericarp. Their contents after storage at 4 °C and at RT were decreased by 22.1–49.7% and 27.6–48.7%, respectively. The oxygen radical absorbance capacity (ORAC) and cellular antioxidant activity (CAA) of litchi pericarp decreased by 17.6% and 58.7% at 4 °C, and by 23.4% and 66.0% at RT, respectively. The results indicated that storage at 4 °C preserved more phenolics and retained higher antioxidant activity in litchi pericarp compared to storage at RT, suggesting that storage at 4 °C should be considered as a more effective method for slowing down the degradation of litchi pericarp phenolics.

## 1. Introduction

Litchi, which originates in China, is now widely cultivated in warm climates around the world. It has become very popular worldwide due to its special taste and flavor [1]. In addition to fresh consumption, litchi can be used to manufacture wine, juice, vinegar, and jelly [2,3]. Litchi pericarp, accounting for approximately 15% of fresh litchi fruits, is the main byproduct of litchi processing [4].

In traditional Chinese medicine, litchi pericarp has been prescribed for its diuretic, analgesic, antitussive, anti-pyretic, and haemostatic properties [5]. Litchi pericarp has been reported to contain a large amount of phenolics, including anthocyanins, procyanidins, flavonoids, lignans, and sesquiterpenes [1,4,6]. However, as shown in many studies on the preservation of litchi fruits [7,8], browning reaction tends to occur shortly after litchi is harvested. Our preliminary experiment further revealed that the total phenolic contents and oxygen radical absorbance capacity (ORAC) activity of litchi pericarp decreased by 86.7% and 81%, respectively, after 12 h of piled outdoor storage without any treatment. In order to get litchi pericarp to retain high-level phenolics, it is necessary to establish a storage method for litchi pericarp that would slow down the degradation of its phenolics. The storage method of litchi pericarp should also be convenient and economical, due to the very limited market value of litchi pericarp as a byproduct of litchi processing. Although various preservation methods of litchi fruit have been investigated in many studies [9,10], none of them have focused on the storage of litchi pericarp in particular. The changes in the phenolic profiles in litchi pericarp during storage are also unknown.

Therefore, the changes in phenolic profiles and antioxidant activity of litchi pericarp during storage at 4 °C for seven days and at room temperature (RT) for 72 h were investigated in the present study. The purpose of this study is (1) to characterize the changes in the content of phenolics and the antioxidant activity during storage of litchi pericarp under different conditions, and (2) to recommend a suitable storage condition under which the phenolic degradation of litchi pericarp could be slowed down effectively.

## 2. Results and Discussion

### 2.1. Changes of Total Phenolic and Procyanidin Content in Litchi Pericarp Stored at Different Temperatures

**Figure 1:**
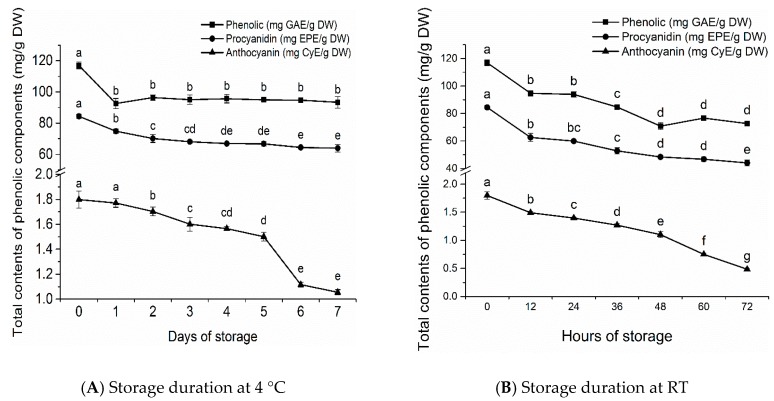
Changes of total contents of phenolic, procyanidin, and anthocyanin in litchi pericarp during storage at 4 °C (**A**) and at room temperature (RT) (27 ± 2 °C) (**B**). Values are expressed as means ± SD, *n* = 3. Different letters at different storage time are significantly different, *p* < 0.05.

During the seven-day storage at 4 °C, the total phenolic and procyanidin contents in litchi pericarp decreased from 116.95 to 93.36 mg gallic acid equivalents (GAE)/g dry weight (DW) and from 84.42 to 63.96 mg epicatechin equivalents (EPE)/g DW, which represented reductions of 20.2% and 24.2%, respectively (*p* < 0.05). As shown in Figure 1A, the total phenolic contents decreased most rapidly by 20.8% (*p* < 0.05) during the one-day storage, and then exhibited insignificant changes (*p* > 0.05) during the subsequent six-day storage. On the other hand, the total procyanidin content decreased by 16.9% after two days (*p* < 0.05). During the subsequent five-day storage, the total procyanidin contents showed a slower decrease from 70.16 to 63.96 mg EPE/g DW.

During the 72-h storage at RT (27 ± 2 °C), the total phenolic and procyanidin contents in litchi pericarp decreased from 116.95 to 72.70 mg GAE/g DW, respectively, and from 84.42 to 44.07 mg EPE/g DW, respectively, representing a decrease of 37.8% and 47.8% (*p* < 0.05), respectively. As shown in Figure 1B, the total phenolic contents decreased most rapidly by 19.0% (*p* < 0.05) during the first 12-h storage. Total phenolic contents exhibited a faster decrease from 93.90 to 70.83 mg GAE/g DW on the second day, which reduced to 60.6% of the fresh sample (*p* < 0.05). However, litchi pericarp maintained stable total phenolic contents on the third day (*p* > 0.05). Total procyanidin contents followed similar trends to those of total phenolics under RT storage. The contents decreased by 25.8% during the first 12-h storage (*p* < 0.05), followed by reduction at a slower rate.

After three-day storage at 4 °C, the total phenolic and procyanidin contents of litchi pericarp reduced to 95.04 mg GAE/g DW and 68.13 mg EPE/g DW, which were 30.7% and 54.6% higher than those stored at RT, respectively. Even after seven days in storage, the total phenolic and procyanidin contents of litchi pericarp were still respectively 28.4% and 45.1% higher than those of the three-day RT storage samples. These indicated that the phenolics in peeled litchi pericarp, including procyanidins, were much more stable at 4 °C than at RT. The phenomenon could be attributed to the faster water loss and higher enzyme activity in the pericarp stored at RT, which accelerated the oxidation of phenolics [11]. The total phenolic and procyanidin contents decreased most rapidly during the first period of the entire storage duration (the first day at 4 °C and the first 12 h at RT, respectively). This could be ascribed to the damage of cell structure of litchi pericarp from the peeling process. Consequently, the polyphenol oxidase (PPO) could interact with phenolics and catalyze their oxidation to quinones, in the presence of molecular oxygen [12]. Similar results have also been reported in strawberries, raspberries, and sour cherries [13]. The total contents of procyanidin decreased more dramatically than those of phenolics both at 4 °C and RT, which could be attributed to the fact that flavan-3-ol monomers and dimers were considered as the primary substrates for enzymatic oxidation [14,15].

### 2.2. Changes of Total Anthocyanin Contents in Litchi Pericarp Stored at Different Temperatures

During the seven-day storage at 4 °C, the total anthocyanin contents in litchi pericarp decreased from 1.80 to 1.06 mg CyE/g DW, representing a reduction of 41.3% (*p* < 0.05). As shown in Figure 1A, the total anthocyanin contents remained unchanged during the one-day storage (*p* > 0.05). However, during the subsequent four days, the total anthocyanin contents decreased from 1.77 to 1.50 mg CyE/g DW (*p* < 0.05), and then to 1.12 mg CyE/g DW after six days of storage (*p* < 0.05). During the 72-h storage at RT, the total anthocyanin contents in litchi pericarp continued to decrease from 1.80 to 0.49 mg CyE/g DW, which represented a reduction of 73.0% (*p* < 0.05).

Anthocyanins have been proven to be the red pigment in litchi pericarp. Although anthocyanins are unstable, they cannot be degraded by PPO or peroxidase (POD) directly, due to the presence of sugar moiety, which causes steric hindrance against enzymatic attack [16]. Anthocyanins of litchi pericarp can be degraded in an anthocyanin–PPO–phenol reaction, in which the degradation rate of anthocyanins can be accelerated by the oxidation products of phenolics [17]. Anthocyanase, which was identified in litchi pericarp, can catalyze anthocyanin hydrolysis to produce anthocyanidins. Anthocyanidins and their degradation products, which have similar structures as catechol, can act as substrates of PPO and POD. Consequently, during the browning of litchi pericarp, anthocyanins can be degraded in an anthocyanase-anthocyanin-PPO [16] or an anthocyanase-anthocyanin-phenolic-H_2_O_2_ reaction [18]. In addition to enzymatic degradation, anthocyanins also undergo non-enzymatic degradation. Ruenroengklin et al. found that pericarp browning was associated with the rapid increase in H_2_O_2_ and ·OH contents of litchi pericarp, and anthocyanins could be degraded substantially in the presence of reactive oxygen species (ROS), particularly hydroxyl radical [19]. This could explain the more dramatic decrease in total anthocyanins than total procyanidins at 4 °C and RT. Similar results have also been reported in strawberries and hawthorn [20,21]. After three days and seven days of storage at 4 °C, the total anthocyanin contents of litchi pericarp were increased by three- and two-fold, respectively, compared to those in the three-day RT storage samples. The observation could potentially be attributed to the higher enzyme activity, greater accumulation of phenolics oxidation products, and additional release of ROS at RT.

### 2.3. Changes of Phenolic Profiles in Litchi Pericarp Stored at Different Temperatures

**Figure 2:**
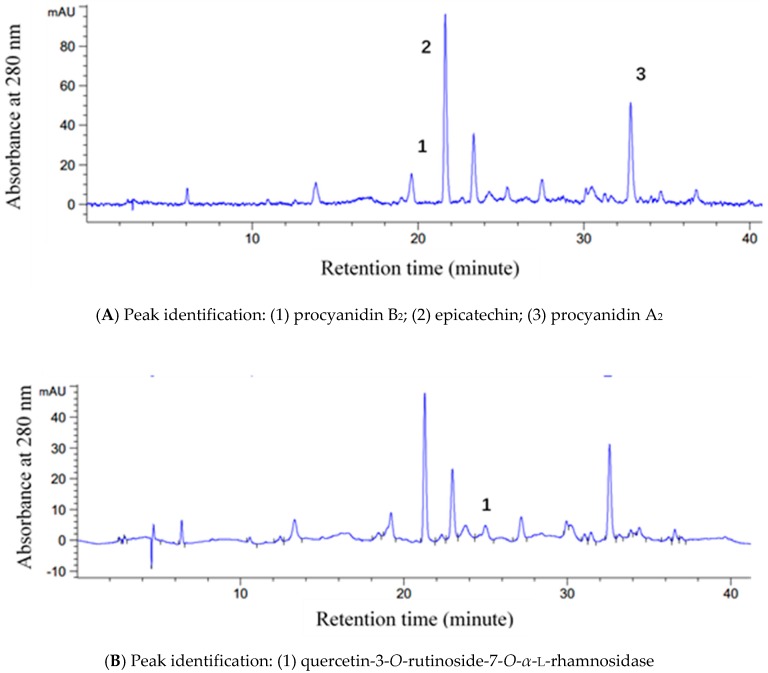
HPLC chromatograms for procyanidin compounds detection (**A**) and flavonoid compounds detection (**B**) in litchi pericarp.

Four phenolic compounds were detected in litchi pericarp (Figure 2), including epicatechin, procyanidin A_2_, procyanidin B_2_, and quercetin-3-*O*-rutinoside-7-*O*-*α*-l-rhamnosidase (QRR), at the levels of 9.83, 17.61, 4.71, and 1.63 mg/g DW in fresh samples, respectively. The changes in the contents of four compounds in litchi pericarp stored at different temperatures were presented in Table 1. During the seven-day storage at 4 °C, the contents of epicatechin, procyanidin A_2_, and procyanidin B_2_ in litchi pericarp continued to decrease, following a similar trend, while the content of QRR decreased slightly faster than those of epicatechin and procyanidin A_2_. The most rapid decrease of QRR was observed on the second day (*p* < 0.05). The contents of epicatechin, procyanidin A_2_, and procyanidin B_2_ decreased faster in litchi pericarp stored at RT than in those stored at 4 °C. The most rapid decreases were observed during the first 12 h of storage (*p* < 0.05), which was consistent with the changes in total procyanidin contents. The contents of QRR decreased most rapidly during the first 12 h of storage at RT (*p* < 0.05), and then remained stable during the subsequent six days of storage.

Flavan-3-ol monomers and dimers have been confirmed as the major substrates of enzymatic oxidation. This could be the reason for the substantial decrease in the flavan-3-ol monomer (epicatechin) and dimers (procyanidin A_2_ and procyanidin B_2_) detected in litchi pericarp during storage at both temperatures. Similar results have been reported regarding the storage of different litchi fruit cultivars [14,15]. B-type procyanidin can be converted into A-type through a free radical-driven oxidative process [22] or an enzyme-catalyzed oxidation reaction [23], indicating that A-type procyanidin may be more stable than B-type procyanidin under conditions that favor oxidative reaction. This could explain the faster decrease in procyanidin B_2_ compared to procyanidin A_2_ during storage at both temperatures. Quercetin glycoside is generally more stable at lower temperatures [24]. As shown in this study, after three-day storage at 4 °C, the QRR contents of litchi pericarp reduced to 1.4 mg/g DW, which was still 18.6% higher than the content of litchi pericarp stored at RT. Furthermore, the litchi pericarp stored at 4 °C for seven days retained more QRR than that stored at RT for three days.

### 2.4. Degradation Kinetics of Phenolics in Litchi Pericarp Stored at Different Temperatures

The contents of litchi pericarp phenolics were fitted to different kinetic models, and the greatest *R*^2^ values were achieved with a first-order reaction model. That is to say, the degradation of litchi pericarp phenolics followed first-order reaction kinetics. However, the changes of total phenolic contents at 4 °C and the contents of procyanidin B_2_ and QRR at RT did not match the model properly (*R*^2^ < 0.7690), which may be attributed to the natural variability of the degradation phenomenon. Similar results have been found in the degradation kinetics of phenolics in hardy kiwifruit [25] and strawberry pulp [26]. The kinetic parameters of phenolics in litchi pericarp stored at different temperatures are shown in Table 2. During 4 °C storage, the *k* value of the procyanidin B_2_ of litchi pericarp was the greatest, followed by that of the total anthocyanidins, and the *k* values of other phenolics were similar. The *t*_1/2_ was defined as the time needed for 50% degradation of phenolics in litchi pericarp. In faster degradation reactions, the higher the *k* values, the shorter the *t*_1/2_ values. These results indicated that the procyanidin B_2_ in litchi pericarp stored at 4 °C degraded fastest, followed by anthocyanidins, and other phenolics showed similar degradation trends. These were consistent with the corresponding decrease of phenolic contents during 4 °C storage. During RT storage, the *k* value of the anthocyanidins of litchi pericarp was the greatest, followed by the epicatechin, procyanidin, total phenolics, and procyanidin A_2_, suggesting that anthocyanidins in litchi pericarp stored at RT degraded fastest, followed by epicatechin, procyanidins, total phenolics, and procyanidin A_2_. This was consistent with the corresponding decrease of phenolics contents during RT storage. In addition, the *k* values were greater and the *t*_1/2_ values were shorter for the litchi pericarp phenolics stored at 4 °C than at RT, indicating that RT storage causes a faster decrease in the contents of litchi pericarp phenolics.

### 2.5. Changes of Antioxidant Activity of Litchi Pericarp Stored at Different Temperatures

**Figure 3:**
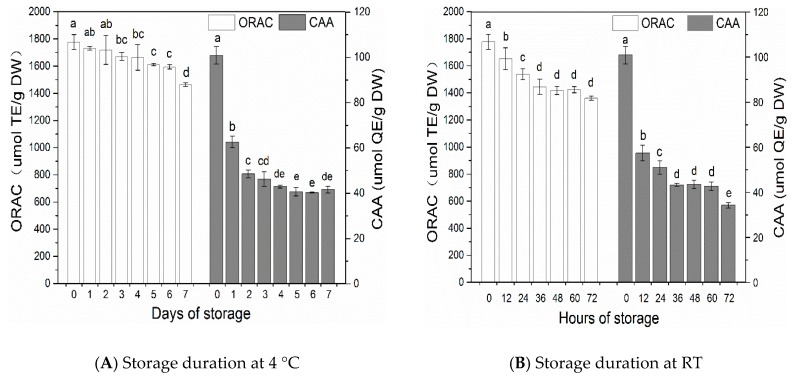
Changes of oxygen radical absorbance capacity (ORAC) and cellular antioxidant activity (CAA) antioxidant activity of litchi pericarp stored at 4 °C (**A**) and at RT (27 ± 2 °C) (**B**). Values are expressed as mean ± SD (*n* = 3). Bars with no letters in common are significantly different, *p* < 0.05.

After seven-day storage at 4 °C, the ORAC and cellular antioxidant activity (CAA) activity of litchi pericarp decreased from 1777.01 to 1463.93 μmol TE/g DW and from 100.89 to 41.67 μmol QE/g DW, which represented reductions of 17.6 % and 58.7 %, respectively (*p* < 0.05). As shown in Figure 3A, the ORAC activity only exhibited a slight reduction after six days of storage, followed by a sharp decrease, from 1593.21 to 1463.93 μmol TE/g DW (*p* < 0.05) on the last day. On the contrary, the CAA activity of litchi pericarp stored at 4 °C decreased most rapidly from 100.89 to 62.59 μmol QE/g DW by 38.0% (*p* < 0.05) on the first day, and then exhibited another rapid decrease from 62.59 to 48.53 μmol QE/g DW (*p* < 0.05) on the second day. The CAA activity of litchi pericarp then decreased slightly during the subsequent five days of storage.

After 72-h storage at RT, the ORAC and CAA activity of litchi pericarp decreased from 1777.01 to 1360.70 μmol TE/g DW and from 100.89 to 34.30 μmol QE/g DW, which represented reductions of 23.4% and 66.0%, respectively (*p* < 0.05). As shown in Figure 3B, the ORAC activity of litchi pericarp continued to decrease by 18.7% (*p* < 0.05) during the first 36 h of storage, and then showed insignificant changes during the remaining time in storage (*p* > 0.05). The CAA activity of litchi pericarp continued to decrease by 57.0% (*p* < 0.05) during the first 36 h of storage, and then remained stable in the subsequent 24 h (*p* > 0.05). However, during the last 12 h, the CAA activity of litchi pericarp exhibited another rapid decrease (*p* < 0.05).

An ORAC assay is based on measuring antioxidant scavenging activity against peroxyl radicals [27]. Strong correlations have been demonstrated between phenolics contents and antioxidant activity in fruits in previous studies [28,29]. In the present study, highly significant correlations were observed between the ORAC activity and the contents of epicatechin, procyanidin A_2_ and procyanidin B_2_ (*r* were 0.871, 0.836 and 0.807 at 4 °C and 0.932, 0.892 and 0.860 at RT, respectively, *p* < 0.01), which were found to be the predominant phenolics in litchi pericarp. Furthermore, the ORAC activity and the QRR contents in litchi pericarp stored at different temperatures also showed significant correlation (*p* < 0.01). These results indicated that flavan-3-ol compounds and QRR might be important contributors to the ORAC activity of litchi pericarp.

The CAA assay, which simulates cellular biological processes, including uptake, metabolism and bioavailability of the antioxidants, is considered to be more biologically relevant than traditional chemical antioxidant assays [30]. A CAA assay is based on detecting the capacity of antioxidants to prevent the formation of fluorescent dichlorofluorescein by 2,2′-azobis (2-amidinopropane) dihydrochloride (AAPH)-generated peroxyl radicals in HepG2 cells. Antioxidants must either bind to the cell membrane to prevent peroxyl radical chain reactions at the cell surface, or enter the cells to react with ROS intracellularly [30]. Correlation analysis showed that the epicatechin, procyanidin A_2_, and procyanidin B_2_ in litchi pericarp were significantly correlative with the CAA activity (*r* = 0.687, 0.870, and 0.8550 at 4 °C, and 0.946, 0.932, and 0.942 at RT, respectively; *p* < 0.01). Likewise, highly significant correlations were also observed between the CAA activity and the contents of QRR (*r* = 0.823 at 4 °C and 0.943 at RT, respectively, *p* < 0.01). It seems that the flavan-3-ol compounds and QRR are important contributors to CAA activity of litchi pericarp. However, Su et al. demonstrated that QRR had relatively higher CAA activity, but epicatechin exhibited lower CAA activity [31]. Therefore, the correlation between epicatechin content and the CAA activity of litchi pericarp might not be a causal relationship. The difference in CAA activity among various phenolic compounds could explain the great discrepancy between the decreasing trends of ORAC and CAA values of litchi pericarp under the same storage conditions. The gap between the CAA values in litchi pericarp was far less than that in the phenolics contents after storage at both 4 °C and RT. This phenomenon indicates that some compounds with a lower stability in litchi pericarp stored at different temperatures might not be contributive to CAA activity.

## 3. Material and Methods

### 3.1. Chemicals and Reagents

Quercetin-3-*O*-rutinoside-7-*O*-*α*-l-rhamnosidase (QRR) was prepared in the laboratory [30]. Procyanidin A_2_ and procyanidin B_2_ were purchased from ChromaDex (Irvine, CA, USA). Epicatechin, gallic acid, quercetin, vanillin, Folin-Ciocalteu reagent, 6-hydroxy-2,5,7,8-tetramethylchroman-2-carboxylic acid (Trolox), fluorescein sodium, 2′,7′-dichlorofluorescin diacetate (DCFH-DA), and 2,2′-azobis (2-amidinopropane) dihydrochloride (AAPH) were obtained from Sigma-Aldrich, Inc. (St. Louis, MO, USA). HPLC-grade glacial acetic acid, acetonitrile, and methanol were obtained from Thermo Fisher Scientific (Suwanee, GA, USA). DMEM(H) medium, new bovine calf serum and Hank’s balanced salt solution (HBSS) were purchased from Gibco Life Technologies (Grand Island, NY, USA). All other reagents were of analytical grade. HepG2 human liver cancer cells were obtained from the American Type Culture Collection (Rockville, MD, USA).

### 3.2. Extraction of Phenolic Compounds form Litchi Pericarp

Fresh litchi fruits cv. Huaizhi were obtained from a local fruit market. Litchi was manually peeled to harvest the pericarp. After washing off the residual pulp and juice with tap water, the litchi pericarp was divided into many portions of 10.00 g each. The weighed litchi pericarp was transferred either to individual sealed polystyrene bags and stored at 4 °C or to uncovered plastic boxes and stored at RT (27 ± 2 °C), avoiding direct sun exposure. To decrease water content for microbial reproduction, the room where litchi pericarp was stored for RT was ventilated with an electronic fan. Therefore, the water content of the samples decreased from 74.61% to 6.43% during the whole storage period. Three portions of litchi pericarp were taken out of storage for analysis every 24 h for those stored at 4 °C until the seventh day, or every 12 h for those stored at RT till the 72nd hour. Then, the phenolics were extracted using a procedure previously described [32], with some modification. Briefly, 10.00 g litchi pericarp was homogenized in an ice bath in 50 mL of 80% (*v*/*v*) chilled aqueous alcohol with XHF-D homogenizer at 5000 rpm for 5 min (Ningbo Xin-zhi-Bio Technology Co. Ltd., Ningbo, China). Another 50 mL of aqueous alcohol was used to clean the homogenizer, and was then collected along with the homogenate. The pooled homogenates were incubated in a sealed flask in a 50 °C water bath for 2 h and then centrifuged at 5000 r/min for 5 min at 4 °C (BIOFUGE, Thermo Fisher Scientific, Waltham, MA, USA). The residue was extracted with 100 mL of aqueous alcohol under the same conditions. The pooled supernatants were concentrated at 45 °C under a vacuum until approximately 90% of the supernatants were evaporated. The concentrated supernatant was then recovered and brought to a final volume of 25 mL with distilled water. The litchi pericarp extracts were stored at −80 °C for further analysis.

### 3.3. Determination of Total Phenolic Contents

The total phenolic contents were determined using the Folin-Ciocalteu method [33]. Briefly, 125 μL of the diluted samples or gallic acid were mixed with 0.5 mL of distilled water in a test tube, and 125 μL of Folin-Ciocalteu regent was subsequently added. The samples were mixed well and let stand for 6 min before 1.25 mL of 7% Na_2_CO_3_ solution was added. Then, water was added to adjust the total volume to 3 mL. After 90 min, the absorbance was measured at 760 nm using a Shimadzu UV-1800 spectrometer (Shimadzu Inc., Kyoto, Japan). Results were expressed as mg gallic acid equivalents (GAE)/g dry weight (DW). All measurements were performed in triplicate.

### 3.4. Determination of Total Procyanidin Contents

Total procyanidin contents were determined according to the sulphuric acid–Vanillin method [34], with some modifications. Briefly, 5 mL mixed solution (3% methanol vanillin solution mixed with equal volume of 30% methanol sulfuric acid solution) was added to 0.5 mL of the appropriately diluted samples or the epicatechin standard solution. The mixture was allowed to stand in the dark for 20 min, and the absorbance was measured at 500 nm using a Shimadzu UV-1800 spectrometer. Results were expressed as mg epicatechin equivalents (EPE)/g DW. All measurements were performed in triplicate.

### 3.5. Determination of Total Anthocyanin Contents

Total anthocyanin contents were measured using a pH-differential method [35]. The samples were separately diluted at the same dilution factor with 25 mM potassium chloride buffer (pH 1.0) or 0.4 M sodium acetate buffer (pH 4.5). Then, absorbance of the diluted samples was measured using a Shimadzu UV-1800 spectrometer at 520 and 700 nm, respectively. The anthocyanin concentrations in the litchi pericarp extracts were calculated as follows:

Anthocyanin concentration (mg cyanidin-3-glucoside equivalents, mg CyE/L) = A × MW × DF × 10^3^/ (ε × 1) (1)

where A is absorbance, and A = (A_520nm_ – A_700nm_)pH1.0 − (A_520nm_ – A_700nm_)pH4.5; MW, the molecular weight of Cy, is 449.2 g/mol; DF is dilution factor of samples; 10^3^ is a factor for conversion from g to mg; ε is the molar extinction coefficient of Cy and is 26,900 L × mol^−1^ × cm^−1^; l is the pathlength in cm. The results were expressed as mg CyE/g DW.

### 3.6. Analysis of Phenolic Profiles

Phenolic profiles of litchi pericarp were analyzed on an Agilent 1200 HPLC system (Waldbronn, Germany), using an Agilent Zorbox SB-C_18_ column (250 × 4.6 mm, 5 μm; Palo Alto, CA, USA). The column temperature was maintained at 30 °C. The mobile phase consisted of 0.4% aqueous solution of acetic acid (solution A) and acetonitrile (solution B). The elution conditions of procyanidins were as follows [36]: 0–20 min, B 5 to 15%; 20–40 min, B 15 to 25%; 40–45 min, B 25 to 35%; and 45–50 min, B 35 to 50%. The elution conditions of flavonoids and phenolic acids were as follows [37]: 0–40 min, B 5 to 25%; 40–45 min, B 25 to 35%; and 45–50 min, B 35 to 50%. Other chromatographic conditions were the same, including an injection volume of 20 μL and a flow rate of 1.0 mL/min. The detection wavelength was set at 280 nm.

Each peak was identified by comparing the retention time with the known authentic standards. Quantitative measurement was performed using the calibration curves of the standards. The results were presented as mg/g DW.

### 3.7. Oxygen Radical Absorbance Capacity Assay

The ORAC assay was conducted using a procedure previously described [27]. The samples and Trolox standard were diluted with 75 mM phosphate buffer (pH = 7.4, working buffer). Then, 20 μL of diluted samples, the Trolox standard (range: 6.25–50 μM), or a working buffer (as a blank) were added to black-walled 96-well plates (Corning Scientific, Corning, NY, USA). Next, 200 μL of 0.96 μM fluorescein was added. The plates were incubated at 37 °C for 20 min on an Infinite M200 Pro plate reader (Tecan Austria GmbH, Salzburg, Austria). Subsequently, 20 μL of 119 mM freshly prepared AAPH was added to each well. The fluorescence intensity was measured at an excitation of 485 nm and an emission of 538 nm every 4.5 min for 35 cycles. The ORAC values were expressed as μmol Trolox equivalents (TE)/g DW. All measurements were performed in triplicate. 

### 3.8. Cellular Antioxidant Activity Assay

The CAA assay was conducted according to a procedure previously described [30]. HepG_2_ cells were seeded in a 96-well microplate (Corning) at a density of 5 × 10^5^/well in 100 μL of growth medium. After 24 h, the growth medium was removed. Cells were then treated with 100 μL of 25 μM DCFH-DA in DMEM containing different concentrations of quercetin or litchi pericarp extractions for 1 h. Each treatment was performed in triplicate. Then, 100 μL of 600 μM AAPH in Hank’s balanced salt solution (HBSS) was quickly added to each treated well. The fluorescence intensity was measured with an Infinite M200 Pro plate reader (Tecan Austria GmbH, Salzburg, Austria) at an excitation of 485 nm and an emission of 538 nm, respectively, every 5 min for 12 cycles. The CAA results were reported as μmol quercetin equivalents (QE)/g DW.

### 3.9. Statistical Analysis

Data were expressed as the mean ± SD. Significance of differences was evaluated by one-way ANOVA followed by the SNK-q test. A value of *p* < 0.05 was used as the threshold for significance. Correlation between variables was determined by Pearson correlation tests. All of the statistical analyses were performed using SPSS 19.0 software (SPSS Inc., Chicago, IL, USA).

## 4. Conclusions

During seven-day storage at 4 °C and 72-h storage at RT (27 ± 2 °C), anthocyanins in litchi pericarp degraded much more quickly than other phenolics including procyanidins. The contents of total phenolics in litchi pericarp decreased more dramatically when stored at RT than at 4 °C. Among four individual phenolic compounds detected in litchi pericarp, epicatechin, procyanidin A_2_, and procyanidin B_2_ continued to decrease following similar decreasing trends, while QRR exhibited the strongest stability at 4 °C and at RT. The contents of the four compounds in litchi pericarp stored at 4 °C decreased much more slowly than those stored at RT. Similar to phenolic contents, the ORAC and CAA activity of litchi pericarp decreased more sharply at RT than at 4 °C. The CAA values decreased most rapidly during the first storage period both at 4 °C and at RT, and CAA activity decreased by more than 50% at the end of storage period at both temperatures. However, the ORAC activities in litchi pericarp stored at 4 °C and at RT remained at 82.4% and 76.6%, respectively. Taken together, more phenolic content and higher antioxidant activity were preserved in litchi pericarp when stored at 4 °C, indicating that this is a more effective storage method for slowing down the degradation of phenolics in litchi pericarp.

## Figures and Tables

**Table 1:** Changes of phenolic compositions and contents in litchi pericarp stored at 4 °C and at RT (27 ± 2 °C) (mg/g dry weight (DW)). Values with no letters in common in each column are significantly different (*p* < 0.05). Values in parentheses indicate decrease percentage to the contents of each individual phenolic in litchi pericarp during storage, respectively.

Time	Epicatechin	Procyanidin A_2_	Procyanidin B_2_	QRR
4 °C				
0 d	9.83 ± 0.17 a	17.61 ± 0.08 a	4.71 ± 0.21 a	1.63 ± 0.03 a
1 d	9.60 ± 0.13 b (2.3)	16.53 ± 0.14 b (6.1)	4.29 ± 0.15 b (8.9)	1.54 ± 0.11 a (5.5)
2 d	9.13 ± 0.03 c (7.1)	15.93 ± 0.07 c (9.5)	3.62 ± 0.21 c (23.1)	1.36 ± 0.07 b (16.6)
3 d	9.00 ± 0.07 c (8.4)	15.4 ± 0.06 d (12.2)	3.27 ± 0.08 d (30.6)	1.40 ± 0.04 bc (14.1)
4 d	8.75 ± 0.03 d (11.0)	14.9 ± 0.15 e (14.9)	3.1 ± 0.05 de (33.1)	1.32 ± 0.08 bc (19.0)
5 d	8.67 ± 0.05 d (11.8)	14.44 ± 0.06 f (18.0)	2.98 ± 0.06 e (36.7)	1.34 ± 0.03 bc (17.8)
6 d	8.16 ± 0.06 e (17.0)	14.29 ± 0.11 f (18.9)	2.94 ± 0.01 e (37.6)	1.28 ± 0.07 c (21.5)
7 d	7.26±0.07 f (26.1)	13.7 ± 0.05 g (22.1)	2.37 ± 0.11 f (49.7)	1.24 ± 0.04 c (23.9)
RT (27 ± 2 °C)				
0 h	9.83 ± 0.17 a	17.61 ± 0.01 a	4.71 ± 0.21 a	1.63 ± 0.03 a
12 h	7.67 ± 0.29 b (22.0)	14.41 ± 0.19 b (18.2)	3.12 ± 0.07 b (33.8)	1.25 ± 0.04 b (23.3)
24 h	6.77 ± 0.17 c (31.1)	13.94 ± 0.20 c (20.8)	2.94 ± 0.07 c (37.6)	1.23 ± 0.01 c (24.5)
36 h	6.13 ± 0.10 d (37.6)	13.64 ± 0.02 cd (22.5)	2.78 ± 0.04 cd (41.0)	1.22 ± 0.01 cd (25.2)
48 h	5.45 ± 0.01 e (44.6)	13.53 ± 0.11 d (23.2)	2.65 ± 0.06 de (43.7)	1.20 ± 0.02 cd (26.4)
60 h	5.18 ± 0.20 ef (47.3)	12.47 ± 0.35 e (29.2)	2.60 ± 0.02 de (44.8)	1.19 ± 0.07 cd (27.0)
72 h	5.04 ± 0.13 f (48.7)	12.09 ± 0.22 f (31.3)	2.47 ± 0.10 e (47.6)	1.18 ± 0.08 d (27.6)

**Table 2:** Degradation kinetic parameters of phenolics in litchi pericarp stored at 4 °C and RT (27 ± 2 °C) as a function of storage time. *k* is the kinetic rate constant (d^−1^); *t*_1/2_ represents the half-life (d); and *R*^2^ is the coefficient of determination.

Compounds	*k* (d^−1^)	*t*_1/2_ (d)	*R* ^2^
4 °C			
Total phenolics	-	-	-
Total procyanidins	0.03468	19.99	0.80
Total anthocyanins	0.07656	9.05	0.82
Epicatechin	0.03713	18.67	0.88
Procyanidin A_2_	0.03344	20.73	0.97
Procyanidin B_2_	0.08724	7.95	0.93
QRR	0.03475	19.95	0.83
RT (27 ± 2 °C)			
Total phenolics	0.1523	4.55	0.82
Total procyanidins	0.1964	3.53	0.88
Total anthocyanins	0.3957	1.75	0.87
Epicatechin	0.2148	3.23	0.91
Procyanidin A_2_	0.1034	6.70	0.80
Procyanidin B_2_	-	-	-
QRR	-	-	-
